# Transcriptional Profiling of the Landes Goose Cecum Following Infection with *Eimeria stigmosa* Isolated from Shanxi Province, North China

**DOI:** 10.3390/microorganisms14081828

**Published:** 2026-08-18

**Authors:** Shuo Li, Ya-Qi Bu, Qian Liu, Zi-Rui Wang, Xing-Quan Zhu, Qing Liu

**Affiliations:** Shanxi Key Laboratory of Animal Disease Research, Prevention and Control, College of Veterinary Medicine, Shanxi Agricultural University, Jinzhong 030801, China; leenianzi@163.com (S.L.); 15832277876@163.com (Y.-Q.B.); l15525635339@163.com (Q.L.); wangzirui8091@126.com (Z.-R.W.); xingquanzhu1@hotmail.com (X.-Q.Z.)

**Keywords:** isolation and identification of *Eimeria* species, *Eimeria stigmosa*, geese, cecum, transcriptome

## Abstract

Though a number of *Eimeria* species have been described in geese, the host responses to these parasites at the molecular level have yet to be explored. In the present study, fresh fecal samples were collected for single-oocyst isolation. The recovered oocysts were identified based on molecular analysis using PCR and sequencing. Subsequently, we examined the transcriptional response of the Landes goose cecum following infection with the isolated *Eimeria* strain. Molecular analysis showed that the isolated *Eimeria* strain was *Eimeria stigmosa*, which was named the *E. stigmosa* SX-01 strain. Transcriptomic profiling identified 3806 differentially expressed genes (DEGs), including 2238 genes with increased expression and 1568 genes with decreased expression. The results obtained from the quantitative reverse transcription PCR (qRT-PCR) analysis confirmed that the RNA sequencing (RNA-seq) data were reliable. According to pathway enrichment analysis for the obtained DEGs, 24 pathways were significantly affected following infection with the *E. stigmosa* SX-01 strain, such as cytokine–cytokine receptor interaction, intestinal immune network for IgA production, cell adhesion molecules, gap junction, arachidonic acid metabolism, alpha-Linolenic acid metabolism, retinol metabolism, and PPAR signaling pathway. These transcriptional alterations were observed in *E. stigmosa*-infected geese without obvious clinical signs, indicating that *E. stigmosa* infection may trigger a strong subclinical host response related to metabolism, immune and inflammatory responses, and intercellular junctional complex-associated processes. Collectively, these findings have implications for better understanding the *E. stigmosa*–goose interactions at the molecular level and provide a foundation for future functional and comparative studies to dissect the pathogenic mechanisms and molecular markers associated with disease resistance, which are expected to inform the design of effective control strategies.

## 1. Introduction

Coccidiosis is caused by obligate intracellular protozoan parasites of the genus *Eimeria*, which are ubiquitous pathogens in the environment [1]. These parasites infect a wide range of avian hosts with strict species specificity [2,3,4,5], targeting distinct segments of the intestinal tract and inducing lesions ranging from mild diarrhea to severe hemorrhagic enteritis [6]. Chicken coccidiosis causes enormous economic damage to the poultry industry [7,8,9,10]. Meanwhile, goose farming has gained popularity and is also an important sector of the poultry industry. While chicken *Eimeria* parasites have been extensively characterized [11,12,13], research on *Eimeria* species infecting waterfowl remains remarkably limited.

In geese, more than 10 intestinal *Eimeria* species have been described, including *Eimeria anseris*, *Eimeria nocens*, *Eimeria stigmosa*, *Eimeria fulva*, and *Eimeria hermani* [14]. Among these, *E. anseris* and *E. nocens* are regarded as highly virulent, capable of inducing fatal hemorrhagic enteritis in young goslings [15,16]. However, even for these two highly virulent species, existing research remains limited and is largely restricted to a narrow scope (primarily encompassing molecular characterization, pathogenicity analysis, and life cycle descriptions) [15,17].

High-throughput RNA sequencing (RNA-seq) has become the tool of choice for investigating host–parasite interactions at the transcriptome level. Using this technology, *Eimeria* infection in chickens was reported to elicit profound transcriptional remodeling of the intestinal mucosa, such as innate immune activation, cytokine signaling, and epithelial barrier disruption [5,18]. To date, the chicken *Eimeria* species investigated included *Eimeria tenella*, *Eimeria maxima*, *Eimeria acervulina*, and *Eimeria necatrix* [19,20,21,22]. Regarding the experimental animals, some studies used chickens with differing susceptibility levels [19,21]. In addition, a recent study revealed cellular responses and state shifts during *E. tenella* infection using single-cell RNA-sequencing analysis of the chicken cecum [23]. However, to the best of our knowledge, no published study has examined the host transcriptional response to any goose-infecting *Eimeria* species. This knowledge gap is particularly significant given that chickens and geese exhibit marked differences in digestive physiological characteristics [24], and that goose *Eimeria* species also differ substantially from chicken *Eimeria* species. For example, the asexual and sexual developmental forms of *E. hermani* and *E. stigmosa* occurred within the nucleus and could be found in the jejunum and ileum of the small intestine [25,26,27]. In addition, the sexual stage of *E. hermani* and *E. stigmosa* also takes place in the cecum [26,27]. Thus, the cecum is a suitable site for assessing the host transcriptional response to sexual development, avoiding confounding transcriptional changes from the preceding schizogony phase.

The present study aimed to characterize the transcriptomic response of the goose cecum to *Eimeria* infection. To achieve this, fecal samples were collected from Landes geese to isolate and identify the *Eimeria* species. Following infection, cecal samples were collected from Landes geese in both infected and control groups for RNA-seq analysis.

## 2. Materials and Methods

### 2.1. Isolation of Pure Eimeria Species

In April 2025, collection of fecal samples from Landes geese was performed in Datong City, Shanxi Province, north China. The fecal samples were mixed with an appropriate amount of tap water, and then filtered sequentially through a 60-mesh sieve and a 260-mesh sieve. The filtrate was collected in a 1000 mL beaker and allowed to stand for 60 min. After discarding the supernatant, the sediment was mixed with 2.5% potassium dichromate and incubated at a constant temperature of 28 °C in an incubator for 5 days, after which, the mixture was transferred into a 50 mL centrifuge tube and centrifuged at 2500 rpm for 10 min. After discarding the supernatant, the saturated saline solution was added. After microscopic examination, the oocysts that floated on the surface were collected.

The oocysts were used to infect geese to obtain large amounts of progeny oocysts. To obtain a pure strain, the progeny oocysts were then subjected to single-oocyst isolation, followed by oral inoculation of geese. At 5.5 days post-infection (dpi), the oocysts were collected and allowed to sporulate.

### 2.2. Identification of Eimeria Species

For molecular identification, the sporulated oocysts were subjected to genomic DNA extraction using a blood/cell/tissue genomic DNA extraction kit (Tiangen Biotech Co., Beijing, China). The internal transcribed spacer (ITS) region of ribosomal DNA was amplified by PCR using the two primers: ITSF (5′-GGATGCAAAACTCGTAACACG-3′), and ITSR (5′-TCCTCCGCTTAATAATATGCT-3′) [28]. The amplification was performed in a 50 µL reaction mixture containing 2 μL of genomic DNA, 0.3 μM of each forward and reverse primer, 25 μL of PrimeSTAR Max Premix (TaKaRa, Dalian, China), and 20 μL of sterile nuclease-free water (Solarbio, Beijing, China). The PCR program consisted of 30 cycles of denaturation at 98 °C for 10 s, annealing at 50 °C for 5 s, and extension at 72 °C for 10 s. PCR products were separated by electrophoresis on 1.5% agarose gels containing ethidium bromide and visualized under UV transillumination. Amplicons of expected sizes were gel purified, cloned into the pMD19-T vector, and sequenced via Sanger sequencing (Sangon Biotech, Shanghai, China).

### 2.3. Experimental Design to Obtain Infected and Healthy Cecal Samples

Six one-day-old male Landes goslings were obtained from a commercial hatchery. All goslings were housed in an isolator and provided ad libitum access to feed and water free of anticoccidials and antibiotics. At 21 days of age, geese were randomly allocated into two groups of three geese each: an infected group named CI, and an uninfected control group named CC. Geese in each group were housed in an individual isolator. Each goose in the CI group was inoculated with 1.5 × 10^4^ sporulated *E. stigmosa* oocysts delivered by oral gavage in a volume of 1 mL sterile PBS. Each goose in the CC group received an equivalent volume of sterile PBS by oral gavage.

### 2.4. Cecal Sample Collection

To determine the effects of the gametogony stage of *E. stigmosa* on the cecum, cecal tissue was collected at 4.5 dpi. Briefly, the geese were humanely euthanized by cervical dislocation, and ceca were opened longitudinally. Following three washes with ice-cold sterile PBS, approximately 200 mg of cecal mucosa was scraped using a sterile scalpel blade, immediately snap-frozen in liquid nitrogen, and transferred to −80 °C storage until RNA extraction.

### 2.5. Library Construction and RNA-Seq

For each group, total RNA was extracted from three biological replicates using the TRIzol reagent (Invitrogen, Carlsbad, CA, USA). Assessment of RNA integrity was carried out using the RNA Nano 6000 Assay Kit of the Bioanalyzer 2100 system (Agilent Technologies, Santa Clara, CA, USA). Poly-T oligo-attached magnetic beads were used to isolate mRNA from total RNA, followed by fragmentation with divalent cations at high temperature. First-strand cDNA was synthesized using random hexamer primers and M-MuLV Reverse Transcriptase, while second-strand cDNA synthesis was performed using DNA Polymerase I and RNase H. After end repair, the 3′ ends of cDNA were adenylated, followed by adaptor ligation and AMPure XP size-selection. Then, the fragments were subjected to PCR amplification, purification of the amplicons, quality control, and cluster generation. Library sequencing was performed on an Illumina NovaSeq platform by Novogene Co., Ltd. (Beijing, China).

### 2.6. Bioinformatics Analysis

After removing reads containing adapters, reads containing poly-N, and low-quality reads from raw data, the clean reads were obtained. Then, Q20, Q30, and GC content of the clean data were calculated. The clean reads were mapped to the domestic goose (*Anser cygnoides domesticus* reference genome (GCF_000971095.1)) using HISAT2 (v2.2.1). The number of reads mapped to each gene was obtained using featureCounts (v2.1.1), and expression levels were normalized as fragments per kilobase of transcript per million mapped reads (FPKM).

Differential expression analysis between the two groups was carried out using the DESeq2 R package (v1.42.0). Genes meeting the dual criteria of |log_2_ fold change| ≥ 1 and a *p*-value < 0.05 were considered as differentially expressed genes (DEGs). Volcano plots were generated using the ggplot2 package in R, and hierarchical clustering heatmaps were generated using the pheatmap package in R (v4.3.3). Gene Ontology (GO) and Kyoto Encyclopedia of Genes and Genomes (KEGG) pathway enrichment analyses were performed using the clusterProfiler R package (v4.8.1), and an adjusted *p*-value < 0.05 was considered significantly enriched.

### 2.7. Validation of RNA-Seq Data by Quantitative Reverse Transcription PCR (RT-qPCR)

To verify the RNA-seq results, RT-qPCR was carried out with SYBR Green Master Mix (Takara, Dalian, China) on the QuantStudio 5 Dx Real-Time PCR System (Thermo Fisher Scientific, Waltham, MA, USA), and three reactions were performed per biological replicate. The thermocycling program involved an initial incubation at 95 °C for 30 s, followed by 40 cycles of 95 °C for 5 s and 60 °C for 30 s. The relative expression levels of the target genes (*LOC106048382*, *LOC106037779*, *PIK3R6*, *SOCS1*, *ATP6V0A4*, *SLC37A2*, *SLC38A4*, and *SLC9A2*) were normalized to those of the internal reference gene (*β-actin*), and the target genes from the infected group were compared with the corresponding target genes from the control group using the 2^−ΔΔCt^ method [29]. Details of the primers are shown in Table S1.

## 3. Results

### 3.1. Molecular Characterization Identified the Species as E. stigmosa

**Figure 1:**
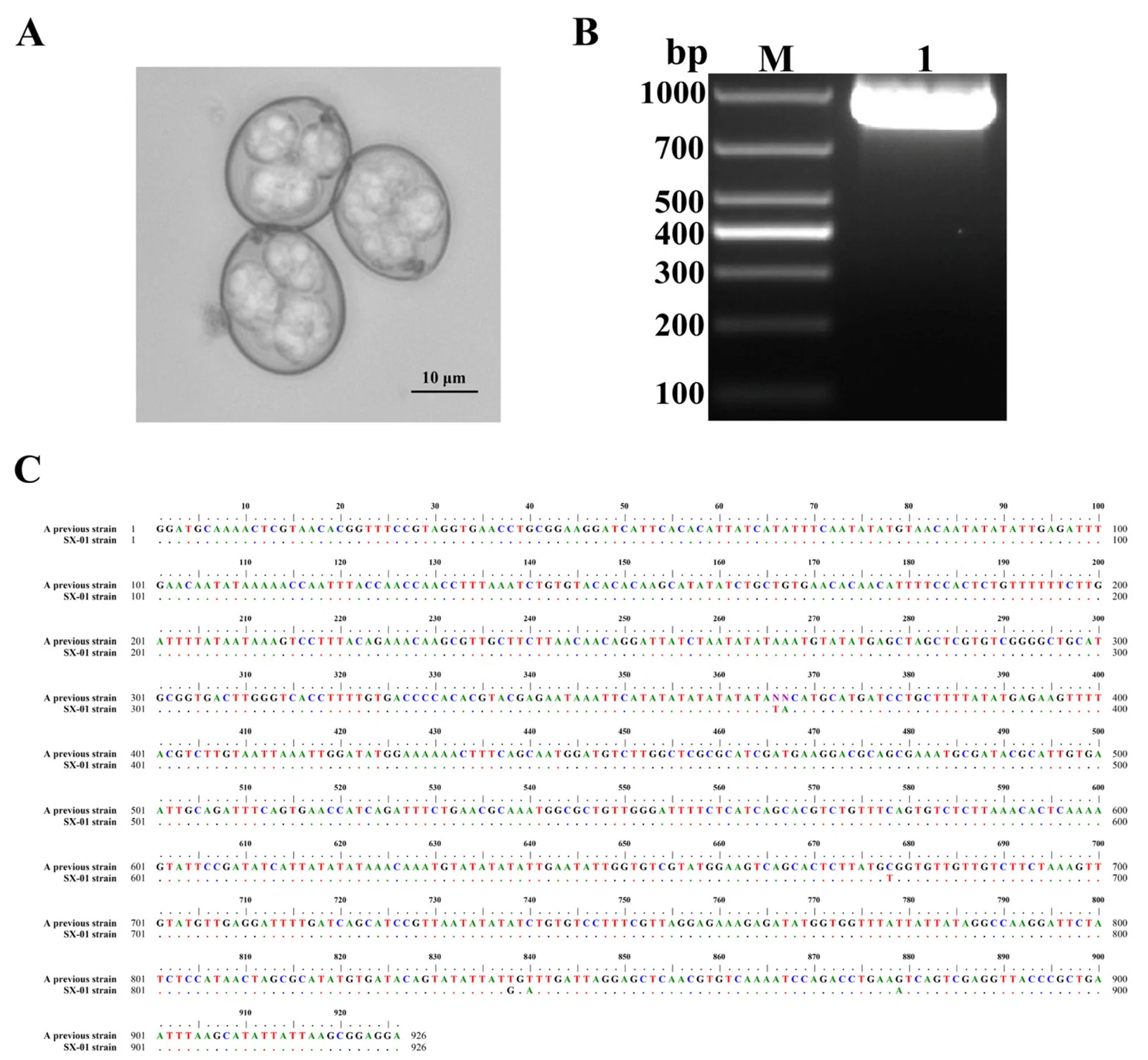
Isolation and identification of an *Eimeria* species infecting Landes geese. (**A**) Mature oocysts were observed using a light microscope. (**B**) Agarose gel electrophoresis of PCR products. Lane M: DNA marker; lane 1: primers targeting the internal transcribed spacer (ITS) region were used to amplify the gene fragment from genomic DNA extracted from oocysts. (**C**) The ITS sequence identified in the present study was aligned with that of a previous *Eimeria stigmosa* strain (GenBank accession No. KP789173.1). Dots represent identical bases.

Single-oocyst isolation was performed, and the oocysts were successfully obtained (Figure 1A). The results of the nested PCR targeting the ITS region showed the presence of a specific band with the size of approximately 900 bp (Figure 1B). Sequencing analysis showed that the ITS sequence of the SX-01 strain obtained in the present study had a high level of sequence identity (greater than 99.3%) with that of a previous *E. stigmosa* strain (GenBank accession No. KP789173.1) (Figure 1C).

### 3.2. RNA-Seq Read Characteristics and Gene Expression Changes

Following adapter trimming and quality filtering, the clean reads obtained per sample ranged from 38,853,208 to 49,461,720 (Table S2). The Q30 scores were observed to be 96.03–97.95%, and the GC content fell within the interval of 47.87–49.28% (Table S2). These findings confirmed the excellent quality of the resulting RNA-seq data.

**Figure 2:**
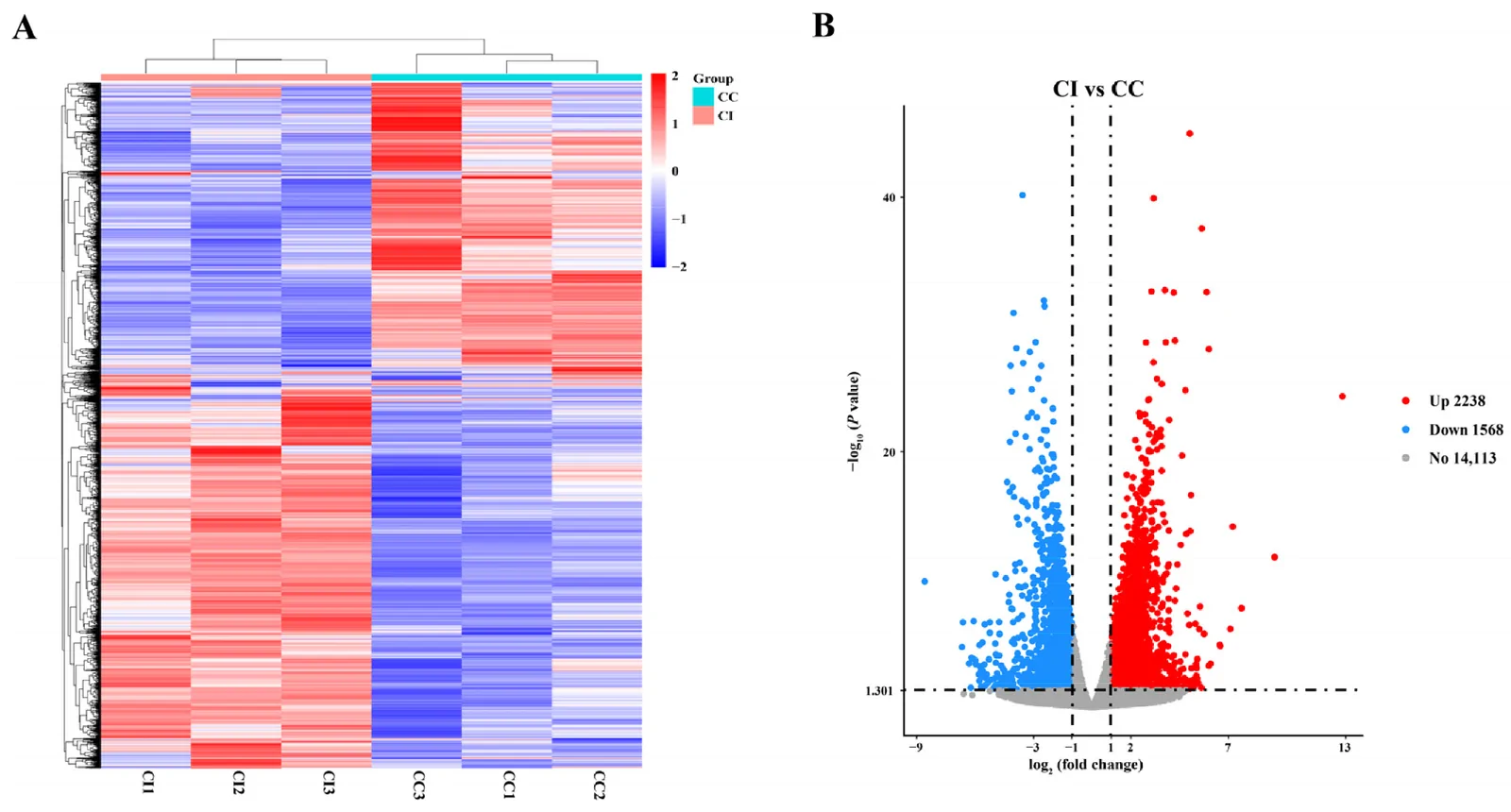
(**A**) Heatmaps highlighting the differentially expressed genes (DEGs) between the infected group and the control group. (**B**) Volcano plot illustrating the DEGs in the infected group compared with the control group. CC: the cecum of uninfected geese; CI: the cecum of *E. stigmosa*-infected geese.

The heatmap showed that the expression of many mRNAs in the CI group was altered compared with that in the control group (Figure 2A). The volcano plot showed that a total of 3806 DEGs were identified, comprising 2238 upregulated and 1568 downregulated genes (Figure 2B) (Table S3).

### 3.3. Validation of DEGs by RT-qPCR

**Figure 3:**
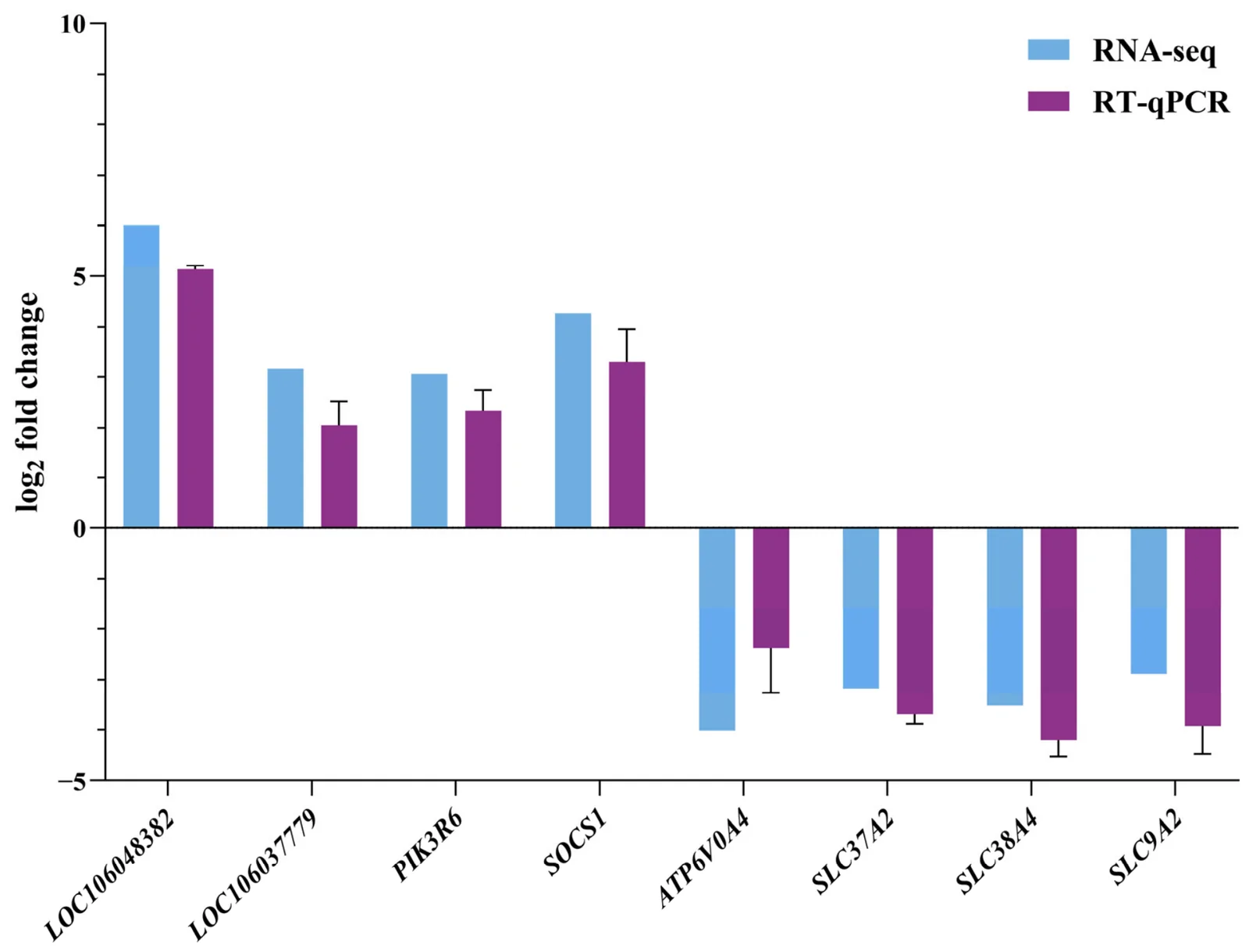
Validation of RNA sequencing (RNA-seq) data by quantitative reverse transcription PCR (RT-qPCR). Eight DEGs (*LOC106048382*, *LOC106037779*, *PIK3R6*, *SOCS1*, *ATP6V0A4*, *SLC37A2*, *SLC38A4*, and *SLC9A2*) obtained from the RNA-seq data analysis were subjected to RT-qPCR analysis. Error bars indicate standard deviations.

To validate the RNA-seq results, the mRNA expression levels of eight DEGs were measured by using the RT-qPCR method. As shown in Figure 3, there was an agreement between the results obtained by RT-qPCR and the RNA-seq data.

### 3.4. GO and KEGG Pathway Enrichment Analyses

**Figure 4:**
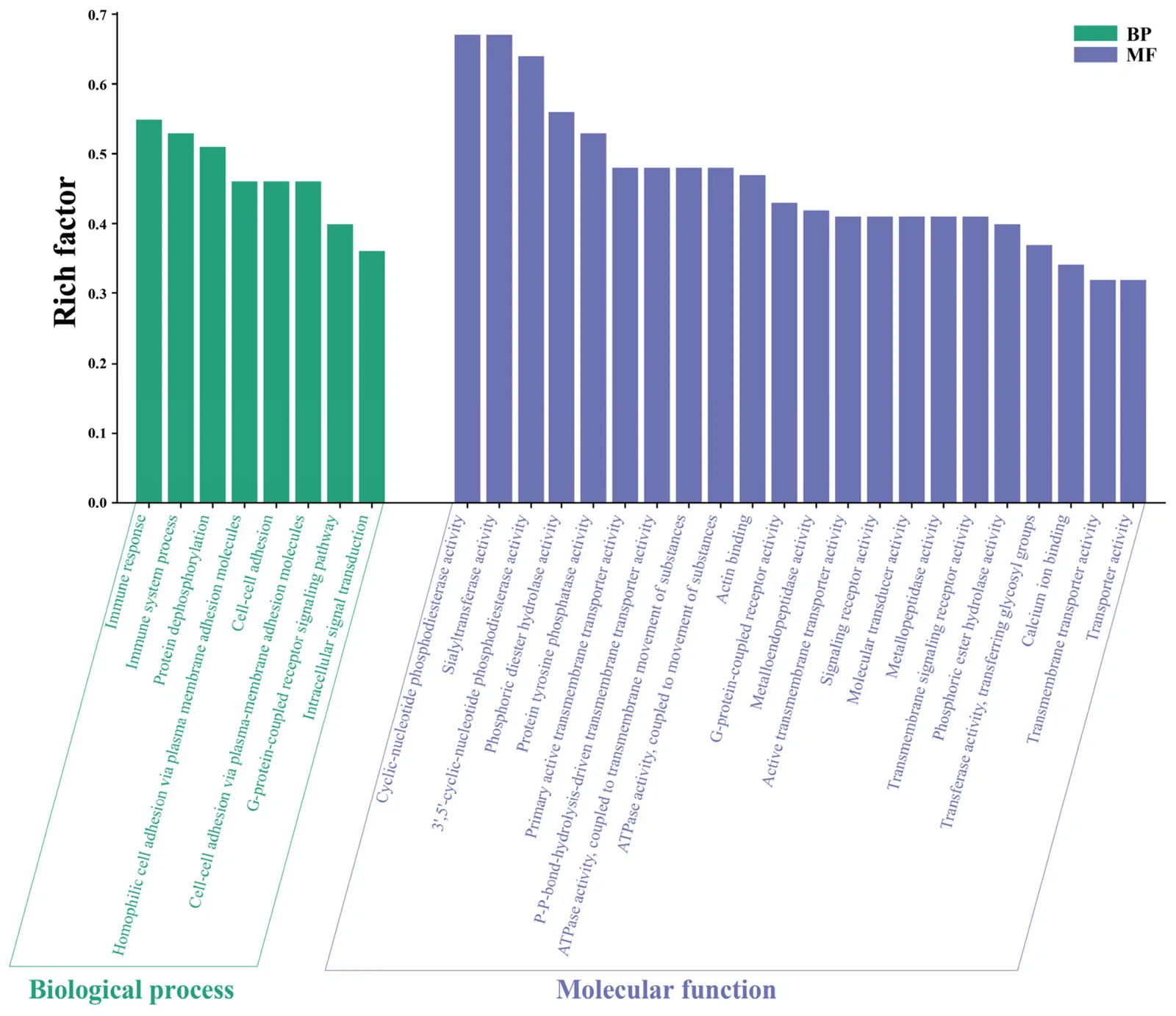
The 30 significantly enriched Gene Ontology (GO) terms for the DEGs between the infected group and the control group, including 8 GO biological process terms and 22 GO molecular function terms.

GO functional enrichment analysis of the identified DEGs yielded a total of 54 enriched GO terms, including 10 and 27 GO terms for biological processes and molecular functions, respectively. The top 30 significantly enriched terms included eight GO biological process terms (G-protein-coupled receptor signaling pathway, intracellular signal transduction, immune response, immune system process, protein dephosphorylation, homophilic cell adhesion via plasma membrane adhesion molecules, cell–cell adhesion, and cell–cell adhesion via plasma-membrane adhesion molecules) and 22 GO molecular function terms (signaling receptor activity, molecular transducer activity, G-protein-coupled receptor activity, transmembrane signaling receptor activity, phosphoric diester hydrolase activity, phosphoric ester hydrolase activity, transporter activity, protein tyrosine phosphatase activity, transmembrane transporter activity, metallopeptidase activity, calcium ion binding, cyclic-nucleotide phosphodiesterase activity, sialyltransferase activity, actin binding, active transmembrane transporter activity, metalloendopeptidase activity, 3′,5′-cyclic-nucleotide phosphodiesterase activity, transferase activity, transferring glycosyl groups, primary active transmembrane transporter activity, P-P-bond-hydrolysis-driven transmembrane transporter activity, ATPase activity, coupled to transmembrane movement of substances, and ATPase activity, coupled to movement of substances) (Figure 4).

**Figure 5:**
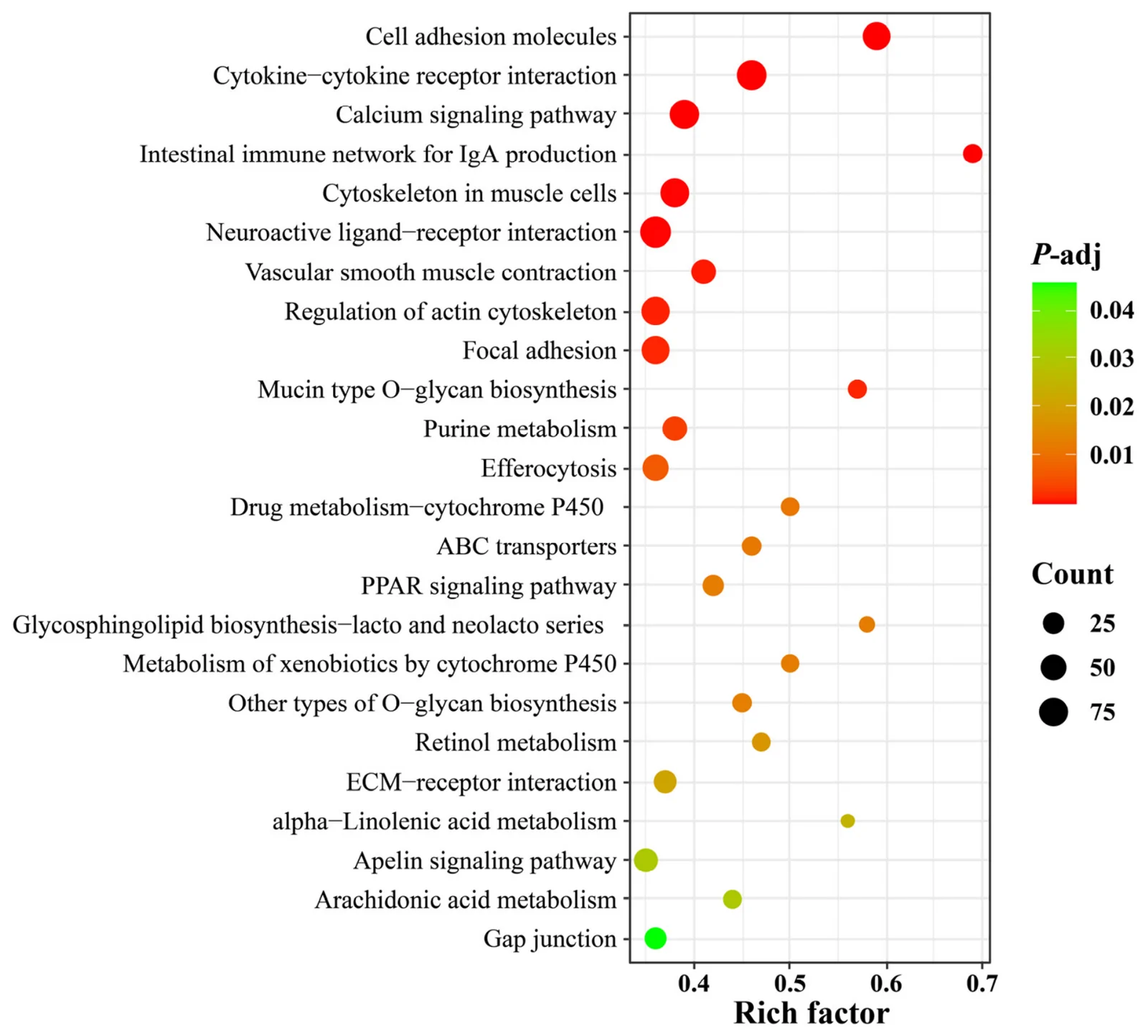
The 24 significantly enriched Kyoto Encyclopedia of Genes and Genomes (KEGG) pathways for the DEGs between the infected group and the control group. The size of each bubble indicates the gene count, while the color gradient indicates the adjusted *p*-value (*P*-adj).

Also, KEGG pathway analysis of transcriptional data was carried out to investigate the cellular functions altered following *E. stigmosa* infection. The DEGs were significantly enriched in 24 pathways, including cytokine–cytokine receptor interaction, intestinal immune network for IgA production, cell adhesion molecules, calcium signaling pathway, gap junction, apelin signaling pathway, arachidonic acid metabolism, alpha-Linolenic acid metabolism, ECM-receptor interaction, retinol metabolism, other types of O-glycan biosynthesis, PPAR signaling pathway, glycosphingolipid biosynthesis-lacto and neolacto series, metabolism of xenobiotics by cytochrome P450, ABC transporters, drug metabolism-cytochrome P450, efferocytosis, purine metabolism, focal adhesion, mucin type O-glycan biosynthesis, regulation of actin cytoskeleton, vascular smooth muscle contraction, neuroactive ligand–receptor interaction, and cytoskeleton in muscle cells (Figure 5).

## 4. Discussion

In the present study, a pure *Eimeria* strain infecting geese was isolated in Shanxi Province, north China. Because the morphological features of sporulated oocysts overlap among different species, molecular identification of the isolated strain was performed by PCR amplification and sequencing analysis. The ITS sequence obtained in this study showed high identity (99.35%) with that of *E. stigmosa* previously reported (GenBank accession No. KP789173.1).

Though *E. stigmosa* does not cause clinical disease or death [14], it may cause alterations in goose intestine at the molecular level. Geese possess a well-developed cecum, which plays an important role in host food digestion, nutrient absorption, and immune regulation [30,31,32]. Meanwhile, the sexual stages of *E. stigmosa* are present in goose cecum between 4 and 5 dpi [25]. Hence, in the present study, we performed a comprehensive transcriptomic analysis of goose cecal tissue at 4.5 dpi in response to the gametogony stage of *E. stigmosa* infection. A total of 3806 DEGs were identified between the infected and control groups, including 2238 upregulated and 1568 downregulated genes. The reliability of RNA-seq data was confirmed by qRT-PCR analysis. To the best of our knowledge, this represents the first transcriptomic investigation of host responses to *E. stigmosa* infection in geese. Given that *E. stigmosa*-infected geese showed no obvious clinical signs, the large number of DEGs identified between the two groups indicated that *E. stigmosa* infection may trigger a strong subclinical host response. This strong response may be due to the intranuclear developmental characteristics of *E. stigmosa* [25]. However, given that changes at the transcriptional level do not always correspond to protein expression, further investigations at the proteomic level are warranted to more comprehensively reveal the host response to *E. stigmosa* infection.

The upregulated genes included *IFNG* (interferon-gamma gene), *IL-10* (encoding interleukin-10), *IRF1* (interferon regulatory factor 1), and *RSAD2* (radical S-adenosyl methionine domain containing 2). Intriguingly, a recent study reported that *IFNG*, *IRF1*, and *IL-10* exhibited significant upregulation in the chicken duodenum following *E. acervulina* infection [20]. Interferon gamma (IFN-γ) plays an important role in innate immune responses against a variety of pathogens [33]. *IRF1* and *RSAD2* are IFN-stimulated genes [34]. The upregulation of *IFNG*, *IRF1*, and *RSAD2* expression indicated a possible role for IFN-γ and IFN-stimulated genes in the innate defense against *Eimeria* replication. The results were consistent with those reported in a previous study, which performed a transcriptome analysis of cecal tissue during primary *E*. *tenella* infection in chickens [35]. During protozoan infections, *IL-10* is thought to regulate the potentially harmful effects that anti-parasite immune responses can inflict on the host [36,37]. Gene expression upregulation of *IL-10* observed in the present study suggested that suppression of anti-parasitic immunity may exist during *E. stigmosa* infection.

Of the significantly enriched GO terms, G-protein-coupled receptor signaling pathway exhibited the largest count of significantly differentially expressed genes. Sphingosine-1-phosphate (S1P), a significant bioactive sphingolipid, is involved in diverse cellular processes by binding to five G-protein-coupled receptors [38]. There are five cell surface G-protein coupled receptors (S1PR1–5) for S1P [39]. In the present study, four S1PRs but not S1PR5 were detected, a finding that was in accordance with the fact that S1PR5 is primarily expressed in the spleen and central nervous system [40]. S1P receptors exhibit overlapping as well as distinct functions [40]. The upregulation of S1PR1–4 in the infected group indicated that *E. stigmosa* infection may affect several biological processes related to S1P receptors. KEGG analysis showed that the significantly enriched pathways included ABC transporters. Since S1P requires transport by ABC transporters to the extracellular space before binding to G protein-coupled receptors to exert its signaling functions [40], this enrichment further indicated that the S1P/S1PRs signaling may be affected during *E. stigmosa* infection.

Also, KEGG analysis showed that some significantly enriched pathways for DEGs were associated with immunity and inflammation, such as cytokine–cytokine receptor interaction, intestinal immune network for IgA production, and PPAR signaling pathway. Cytokine–cytokine receptor interaction and intestinal immune network for IgA production were commonly found in chickens infected with *Eimeria* species [41,42]. Previous studies showed that the removal of the bursa by chemical or hormonal means does not diminish the development of protective immunity against *Eimeria* reinfection [43], and IgA production was thought to be unimportant in host defense against chicken coccidiosis. Hence, cytokine–cytokine receptor interaction may be critical for controlling *E. stigmosa* infection. However, it was not until 2026 that a study provided the evidence for a Bursa of Fabricius-independent B-cell developmental pathway and its functional role in sustaining intestinal IgA production [44]. Thus, the critical roles of IgA production for controlling *E. stigmosa* infection should not be excluded. Nevertheless, given that *E. stigmosa*-infected geese displayed no obvious clinical signs, these immune pathways alone may be insufficient to confer complete protection. The intercellular junctional complex in epithelial cellular sheets includes focal adhesions, tight junctions, gap junctions, adherens junctions, and desmosomes, which plays an essential role in preserving barrier integrity, enabling cell adhesion, mediating the transfer of ions and metabolites, and supporting connections both between cells, and between cells and the extracellular matrix [45]. In the present study, focal adhesion and gap junction were significantly enriched for the DEGs, indicating that the processes related to the intercellular junctional complex may play important roles in controlling *E. stigmosa* infection.

In addition, several metabolism-related pathways were significantly enriched, including drug metabolism-cytochrome P450, purine metabolism, retinol metabolism, and arachidonic acid metabolism. Notably, some metabolism-related pathways were closely linked. In particular, drug metabolism-cytochrome P450 mediates arachidonic acid metabolism [46]. More importantly, the four metabolism-related pathways are associated with immunity and inflammation [46,47,48]. These findings indicated that metabolic regulation of the immune system may exist to control *E. stigmosa* infection.

The present study has several limitations. For example, the number of biological replicates per group was three, which represents the minimum acceptable threshold. In addition, our analysis was confined to a single goose-infecting *Eimeria* species and a single time point. Future investigations incorporating additional *Eimeria* species and a broader temporal scope are warranted to better understand host–parasite interactions.

## 5. Conclusions

In summary, a pure *E. stigmosa* strain was firstly isolated in Shanxi Province, north China. The present study revealed, for the first time, the gene expression changes in the goose cecum during *E. stigmosa* gametogony; the mRNA expression level of 3806 genes in the goose cecum was significantly altered following *E. stigmosa* infection, including 2238 upregulated transcripts and 1568 downregulated transcripts; *E. stigmosa* infection may affect several aspects of the goose cecum, including metabolism, immune and inflammatory responses, and intercellular junctional complex-associated processes. These findings provide a basis to better understand the goose–*E. stigmosa* interactions at the molecular level. The findings also form the basis for functional and comparative studies to pinpoint the biological processes underpinning pathogenic mechanisms and support the design of control strategies to reduce economic losses and safeguard food safety.

## Data Availability

The raw data of RNA-seq transcriptome datasets in the present study was submitted to the NCBI Sequence Read Archive (SRA) under BioProject accession number PRJNA1484413.

## Supplementary Materials

The following supporting information can be downloaded at https://www.mdpi.com/article/10.3390/microorganisms14081828/s1, Table S1: Primers used for RT-qPCR analysis; Table S2: Summary of RNA-seq data; Table S3: The DEGs obtained in the present study.
