# Supplementary material for: Transcriptional Profiling of the Landes Goose Cecum Following Infection with Eimeria stigmosa Isolated from Shanxi Province, North China
Source: Microorganisms. 2026 Aug 18;14(8):1828. doi: 10.3390/microorganisms14081828 (PMC13515907; doi:10.3390/microorganisms14081828)
Supplement: Supplementary file 1 [file microorganisms-14-01828-s001.zip › Table S1.pdf]

Table S1. Primers used for RT-qPCR analysis.

Gene	Forward primer (5′-3′)	Reverse primer (5′-3′)
β-actin	GCACCCAGCACGATGAAAAT	GACAATGGAGGGTCCGGATT
LOC106048382	CAAACCCGTGCACTTCACAG	TGCTCTCCCGATCTCACTGA
LOC106037779	CCCGATGTACCCTCTGCTTC	TCCAGAAGGCAGCTGTGAAG
PIK3R6	CTCAGGTGATGGTGTGTGCT	GTTTGCTGTGCAGAACCCAG
SOCS1	TCAGTGCTGCCCATGAGAAG	TCTCTTTGCTGCCATCCAGG
ATP6V0A4	TACATTGTCTGGGCTGGCTG	GATCAGATGCGGAGGACCAG
SLC37A2	GAGTTCTCCCTTTGCCTGCT	AATGCGCGACATTCACGATG
SLC38A4	AACGTCAGGCACTTCTGGAG	ACTGGATAGGGTGCTTTGCC
SLC9A2	TTGGGCTATGCACTCCAAGG	GTGCACAGTGACTGCAACTG
