# Supplementary material for: Transcriptional Profiling of the Landes Goose Cecum Following Infection with Eimeria stigmosa Isolated from Shanxi Province, North China
Source: Microorganisms. 2026 Aug 18;14(8):1828. doi: 10.3390/microorganisms14081828 (PMC13515907; doi:10.3390/microorganisms14081828)
Supplement: Supplementary file 1 [file microorganisms-14-01828-s001.zip › Table S2.pdf]

Table S2. Summary of RNA-seq data. CC: the cecum of uninfected geese; CI: the cecum of *Eimeria stigmosa*-infected geese.

**Sample**	**Clean reads**	**Q30 (%)**	**GC (%)**	**Mapping rate (%)**
CC1	42,504,778	97.36	47.87	83.33
CC2	49,461,720	97.95	48.11	82.95
CC3	41,935,038	97.30	47.94	80.90
CI1	40,566,238	97.29	49.28	80.65
CI2	38,853,208	96.03	48.20	80.65
CI3	41,763,046	97.32	48.45	81.07
